# The leucocyte nadir, a predictor of chemotherapy efficacy?

**DOI:** 10.1038/sj.bjc.6690583

**Published:** 1999-08

**Authors:** S Kvinnsland

**Affiliations:** The Norwegian Radium Hospital, Oslo, Montebello, N-0310, Norway


*British Journal of Cancer* (1999) **80**(11), 1681
© 1999 Cancer Research Campaign
Article no. bjoc.1999.0583

Editorial

### The leucocyte nadir, a predictor of chemotherapy efficacy?

**S Kvinnsland**

The Norwegian Radiumhospital, Montebello, N-0310 Oslo, Norway

Among many challenges in chemotherapy of cancer diseases, overtreatment remains one of the greatest. A substantial proportion of patients, perhaps the majority, do not benefit from the chemotherapy that they are offered, either in the adjuvant or in the palliative situation. In the adjuvant situation this is particularly serious, as the same patients will often experience the side-effects following the therapy. For such patients, there is no immediate way to assess the value of therapy.

The response of cancer cells to chemotherapy can simplistically be illustrated as related to two basic, pharmacological characteristics. The first is the accessibility of the therapeutic principle to the cancer cell: the drug in its active form has to reach the target, the malignant cell, wherever it is located. The second is the inherent sensitivity in the target cells, the cancer cells, to the treatment principle. This sensitivity results from a combination of genetic predisposition and acquired resistance. The acquired resistance could be due to previous exposure to toxic substances or be a characteristic from malignant evolution. The sensitivity versus resistance, or the expression of this relation in a tumour cell population, seems to be related to cell proliferation.

If the sensitivity of tumour cells to therapeutic principles is a reflection of the genetic predisposition, this sensitivity would theoretically be the same in all cells in an individual. If, on a given schedule of chemotherapy, the leucocyte nadir is low, this could then indicate more adequate exposure of drug to the tumour cells (and all other cells) in that individual.

In addition to the individual sensitivity-based genetic predisposition, tumour responsiveness to specific drugs, such as anthracyclines and cisplatinum, is probably also related to genetic changes in the tumour cells. Examples are p53 mutations, which seem to indicate lack of response to CMF and doxorubicin (Aas et al, 1996) while the opposite is indicated for cisplatinum and paclitaxel (Hawkins et al, 1996).

The hypothesis of an individual genetic predisposition is tested by Poikonen et al (1994). In patients with primary, node-positive breast cancer and who were given CMF as adjuvant chemotherapy, they conclude that leucocyte nadir is associated with favourable distant disease-free interval and that it could be useful as a biological marker of chemotherapy efficacy. This is a follow-up and confirmation of similar work from the same group (Saarto et al, 1997). In this previous paper, based on results from 211 stage II and III breast cancer patients, it was concluded that a low leucocyte nadir after another, anthracycline-containing adjuvant regimen predicated both prolonged distant disease-free interval and overall survival. The interesting concept of a higher biological dose intensity connected with low leucocyte nadirs and good prognosis compared to high dose intensity due to lower sensitivity (higher leucocyte nadir) is discussed.

Pointing in the same direction are studies on the effect on outcome of dose reductions. In a well conducted, although retrospective, study Colleoni et al (1997) found that patients who had major dose reductions based on severe neutropenia experienced less benefit from the treatment.

If the results in the papers presented by the Finnish group (Saarto et al, 1997; Poikonen et al, 1999) are supported by further documentation, it would support the concept of tailored, individual dosing based on individual sensitivity, best characterized by the leucocyte nadir after the first challenges with chemotherapy. An unresponsive bone marrow after initial standard dose indicates that the treatment is not well tolerated, but too well tolerated. This hypothesis should be tested in a prospective, randomized trial where the patients in the control arm of the trial are given chemotherapy according to traditional conventions, while the patients in the experiment are given individualized, tailored doses of chemotherapy according to bone marrow (leucocyte nadir) response. Perhaps the Finnish investigators will do this?

### REFERENCES

Aas T, Børressen A-L, Geisler S, et al (1996) Spesific p53 mutations are associated with de novo resistance to doxorubicine in breast cancer patients. *Nat Med* **2**: 811–814

Colleoni M, Pricer K, Catiglione-Gertsch M et al (1998) Dose-response effect of adjuvant cyclophosphamide, methotrexate, 5-fluorouracil (CMF) in node-positive breast cancer. *Eur J Cancer* **34**: 1693–1700

Hawkins D, Demers G and Galloway D (1996) Inactivation of p53 enhances sensitivity to multiple chemotherapeutic agents. *Cancer Res* **56**: 892–898

Poikonen P, Saarto T, Lundin J, Joensuu H and Blomquist C (1999) Leucocyte nadir as a marker for chemotherapy efficacy in node positive breast cancer treated with adjuvant CMF. *Br J Cancer* (in press)

Saarto T, Blomquist C, Rissanen P, Auvinen A and Elomaa I (1997) Haematological toxicity: a marker of adjuvant chemotherapy efficacy in stage II and III breast cancer. *Br J Cancer* **75**: 301–305

*Received 3 February 1999*
*Accepted 7 April 1999*

